# Visible–Infrared Fusion Based on CNN and Deformable Transformer

**DOI:** 10.3390/jimaging12060219

**Published:** 2026-05-22

**Authors:** Xiaoyi Wang, Xiansong Gu, Bin Li, Mingqiang Zhang, Panpan Yang, Qiang Fu

**Affiliations:** 1 College of Opto-Electronic Engineering, Changchun University of Science and Technology, Changchun 130022, China; 2 State Key Laboratory of Dynamic Optical Imaging and Measurement, Changchun Institute of Optics, Fine Mechanics and Physics, Chinese Academy of Sciences, Changchun 130033, China

**Keywords:** infrared and visible image fusion, deformable transformer, object detection

## Abstract

To address the limitations of traditional methods in feature extraction and multi-modal information fusion, this paper proposes an infrared–visible image object detection architecture that integrates Convolutional Neural Networks (CNNs) and Deformable Transformers. This method leverages the advantages of CNN in local feature modeling and the capabilities of Transformer in capturing global contextual information, facilitating the fusion of semantic consistency and structural details across modalities. By introducing a detection-aware multi-task optimization mechanism, the model improves object detection in challenging scenarios such as low-light conditions, occlusion, and complex backgrounds. Experiments on multiple standard datasets, including M3FD and LLVIP, indicate that the proposed method achieves competitive or better performance than the compared methods in key metrics such as mAP. Specifically, our method obtains the best mAP50 among the evaluated methods with an mAP50 of 74.2% on the M3FD dataset and 98.6% on the LLVIP dataset, surpassing the second-best PIAFusion by 4.3% and 2.5% respectively. These quantitative results support the practicality and effectiveness of our approach in the evaluated complex environments.

## 1. Introduction

With the rapid development of intelligent perception technology, object detection, as a core task in computer vision, has demonstrated broad application prospects in critical scenarios such as autonomous driving, security surveillance, and intelligent robotics. However, in complex and variable real-world environments, object detection based on single-modal images often faces numerous challenges. For instance, under insufficient illumination, backlighting, or harsh weather conditions, the quality of visible light images degrades severely, leading to a significant deterioration in detection performance. In contrast, infrared images capture the thermal radiation information of objects, possessing strong anti-interference capabilities, making them particularly suitable for extreme conditions like low-light or smoke [1].

Nevertheless, infrared images themselves suffer from low resolution and insufficient detail, making it difficult to provide complete texture and structural information. Therefore, how to effectively fuse infrared and visible light images to exploit their complementary characteristics, thereby enhancing the accuracy and robustness of object detection, has become a focal and challenging issue in current multi-modal perception research.

In recent years, academia has proposed various methods for the task of infrared–visible image fusion for object detection. Early research often relied on pixel-level or feature-level fusion strategies, such as image fusion techniques based on wavelet transform or multi-scale transform, or feature fusion methods based on sparse representation or non-negative matrix factorization. However, these methods predominantly depend on handcrafted feature extractors, exhibit poor adaptability, struggle to cope with modal variations in complex scenes, and generally employ simplistic fusion strategies that fail to fully exploit the semantic complementarity between modalities, limiting the improvement in object detection performance. With the widespread application of deep learning techniques, CNN-based image fusion methods have gradually become mainstream. CNNs can automatically learn multi-level feature representations of images, to some extent improving the discriminability and adaptability of fused images. However, traditional CNNs cannot inherently model global contextual information in images and struggle to capture long-range dependencies, limiting their expressive power in complex scenes. To address this issue, some studies introduced attention mechanisms to enhance inter-modal feature selection and fusion capabilities, but they suffer from high computational costs, and the semantic consistency of the fusion results remains difficult to guarantee.

In recent years, the Transformer architecture, as a powerful sequence modeling tool, has been gradually introduced into computer vision tasks due to its outstanding achievements in natural language processing, demonstrating excellent performance in tasks such as image classification and object detection. Particularly, Deformable Transformer, as a variant, incorporates deformable convolution operations, enabling the model to better adapt to geometric deformations and structural irregularities in images, thereby enhancing the spatial perception capability for object detection. However, how to effectively combine CNN with Deformable Transformer to balance local texture feature extraction and global context modeling remains a critical technical challenge requiring breakthroughs. Furthermore, existing infrared–visible image fusion methods have made progress in improving visual quality, such as enhancing image contrast and preserving structural information, but their performance in downstream tasks like object detection still shows significant shortcomings [1]. The fundamental reason lies in the lack of task-specific optimization for detection in these methods, leading to a mismatch between the feature representation of the fused image and the requirements of the detector, thereby affecting detection performance. Specifically, current methods mainly suffer from three key problems: (1) Feature Incompatibility: Traditional fusion methods struggle to simultaneously retain thermally salient regions in infrared images and detailed textures in visible images. The generated fusion features lack discriminability and are difficult to be effectively utilized by modern detection networks. For instance, methods like DenseFuse [2] and IFCNN [3] focus on general visual quality but often fail to produce features that optimally highlight targets for detectors, as noted in task-oriented fusion studies [4]. (2) Semantic Misalignment: Existing methods mostly employ uniform fusion strategies, neglecting the dependence differences in different target categories on modal features. For example, pedestrian detection relies more on thermal features, while vehicle detection relies more on texture features, leading to inefficient utilization of fused information. Recent benchmarks and analyses highlight that naive fusion without considering semantic guidance results in suboptimal performance for specific object classes [5,6]. (3) Architectural Disconnection: In the mainstream “fuse-then-detect” two-stage approach, the fusion module and the detection module are trained independently, lacking task-cascaded optimization. The fused features may not align with detection requirements, creating an information bottleneck that limits overall performance improvement [7,8,9]. This disconnection prevents the fusion process from being directly guided by the ultimate detection objective.

**Figure 1:**
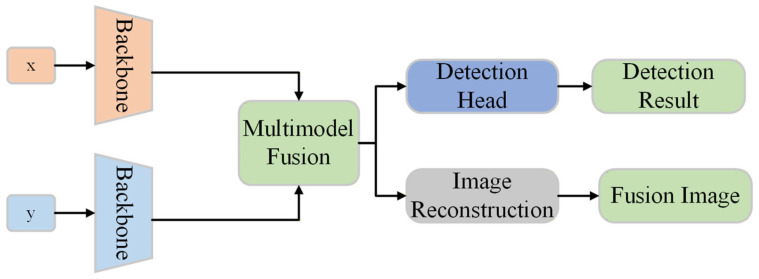
Multi-stages (joint cascaded).

Based on the above problems and the foundational work of our team [10,11,12,13,14], this paper proposes a detection-aware multi-modal fusion detection framework that integrates CNN and Deformable Transformer structures, constructs an end-to-end joint optimization mechanism, and simultaneously introduces a dynamic modal weighting strategy and a multi-spectral pre-training method to enhance modal fusion capability and model generalization performance. This research aims to alleviate the key challenges of poor compatibility, semantic misalignment, and architectural disconnection in current fusion detection methods and provides a practical architectural reference for multi-modal object detection in scenarios such as autonomous driving and intelligent surveillance. As shown in Figure 1, this paper proposes a multi-stage joint cascaded (MJC) detection-aware fusion framework, partially alleviating the performance bottleneck of multi-modal image fusion in object detection. This framework integrates multiple modules, including feature extraction, modal fusion, object detection, and image reconstruction, establishing a unified end-to-end optimization process that supports the joint optimization of fusion and detection tasks.

The main contributions of this study can be summarized as follows:

(1) **Detection-Aware End-to-End Fusion Framework**: We propose an end-to-end optimized fusion architecture that is explicitly aware of the downstream detection task. By establishing a direct gradient pathway between the detection head and the fusion module, the framework enables joint training of the fusion and detection processes. The backpropagation of the detection loss to the fusion layers encourages the model to prioritize features relevant to object recognition during multi-modal integration, thereby implementing a task-driven feature selection mechanism. This approach effectively mitigates the information disconnection inherent in the traditional decoupled “fuse-then-detect” pipeline.

(2) **Dynamic Fusion via Interactive Deformable Attention**: We design a dynamic fusion module built upon an Interactive Deformable Attention mechanism. Leveraging the adaptive receptive field of deformable attention and its powerful capacity for modeling irregular structures, this module facilitates deep cross-modal interaction. It dynamically adjusts the contribution of thermal radiation features from infrared images and texture details from visible images based on the local semantic context, achieving precise and target-oriented feature complementarity.

(3) **Detection-Guided Two-Stage Training Strategy**: We integrate the detection objective into the established two-stage training paradigm. In the first stage, the network learns to decouple and reconstruct cross-modal features, laying a foundation for robust representation. In the second stage, the feature extractor is frozen, and the fusion module is fine-tuned jointly with the detection head under the guidance of the detection loss. This strategy shifts the learning goal from mere “feature reconstruction” toward “task-oriented fusion,” ensuring training stability and making the fusion mechanism explicitly goal-directed, as inspired by related feature decomposition works.

## 2. Related Work

### 2.1. Infrared and Visible Image Fusion

Infrared and visible image fusion aims to integrate information from images acquired by two different sensors, generating a high-quality fused image containing salient target features and rich texture details. In recent years, deep learning methods have made significant progress in the field of image fusion, particularly in enhancing the quality and information content of fused images [15].

**CNN-based Fusion Methods:** Convolutional Neural Networks (CNNs), by virtue of their powerful feature extraction capabilities, have been widely used in infrared–visible image fusion [16]. For example, some studies employ dual-branch CNN structures to extract features from infrared and visible images separately, then generate the final fused image through fusion strategies [17]. To overcome the limitation of the CNN receptive field, researchers combine multi-scale transform (MST) with CNN to obtain more comprehensive image information [16]. PFCFuse [18] proposed a network combining Pool former and CNN for fusion, aiming to more fully utilize valuable information from multi-modal source images to achieve high-quality fusion. DDRF [17] proposed a Dual-branch Decomposition and Reconstruction Fusion (DDRF) architecture, utilizing residual XCiT blocks to extract shallow features from modalities, and then fusing them using a proposed adaptive feature fusion module, aiming to extract meaningful features from the modalities fully.

**Transformer-based Fusion Methods:** The Transformer architecture, with its ability to capture long-range dependencies in images, shows great potential in the field of image fusion. Some studies introduce the Transformer into CNN-based fusion networks to enhance the network’s perception of global information [19]. For instance, MFT [20] proposed a Multi-scale Fusion Transformer to capture global contextual information in images better, thereby improving the quality of the target image. SePT [15] proposed a Semantic-aware infrared and visible image fusion Transformer (SePT), extracting local features through a Convolutional Neural Network (CNN). DATFuse [21] proposed a Dual Attention Transformer, aiming to generate composite images that simultaneously contain the thermal radiation information from infrared images and the rich texture details from visible images, enabling the detection of targets under various weather conditions in high-spatial-resolution scenarios. SFPFusion [15] proposed an improved vision Transformer incorporating super feature attention and wavelet-guided pooling for infrared and visible image fusion, aiming to generate a single image that preserves complementary features and reduces redundant information from different modalities. Conti-Fuse [22] proposed a Continuous Decomposition Fusion framework, better presenting the common and unique features of source modalities through a continuous decomposition strategy.

**CNN–Transformer Hybrid Methods:** To balance local feature extraction and global information modeling, some studies attempt to combine CNN with Transformer [23,24,25]. For example, HDCCT [23] proposed a Hybrid Densely Connected CNN and Transformer (HDCCT) fusion framework, aiming for better feature representation. HDCTfusion [24] proposed a dual-branch fusion network combining Convolutional Neural Network (CNN) and Transformer, aiming to fuse target information with rich details and contrast. FDB-Net [26] proposed a Fusion Dual Branch network combining CNN and Transformer for medical image segmentation.

### 2.2. Multi-Modal Object Detection

Multi-modal object detection aims to utilize data acquired by multiple sensors to improve the accuracy and robustness of object detection. In complex scenes, images from a single modality may suffer from information loss or noise interference, while multi-modal image fusion can effectively compensate for these deficiencies [27].

**Application of Infrared–Visible Image Fusion in Object Detection:** Infrared images are sensitive to thermal radiation and can effectively detect targets in low-light environments; visible light images provide rich texture details, facilitating precise target localization. Fusing infrared and visible images can significantly enhance object detection performance [28]. For instance, some studies use fused images as input to object detection models, thereby improving the recognition ability of targets in complex scenes [26].

Deep Learning-based Multi-modal Object Detection Methods: Deep learning techniques provide new solutions for multi-modal object detection. Some researchers utilize deep learning networks to learn feature representations of multi-modal images and integrate features from different modalities through fusion strategies [29]. For example, one study proposed a feature-level fusion-based infrared–visible image vehicle target detection method, which first obtains registered infrared–visible image pairs, extracts image features separately, and then performs fusion detection [28]. Enhanced Small Target Detection [30] proposed a small target detection method based on multi-modal image fusion and attention mechanisms. This method utilizes YOLOv5, integrating infrared and visible images to enhance the detection of small targets in complex environments. CTAFFNet [31] proposed a new Convolutional Neural Network (CNN)–Transformer Adaptive Feature Fusion Network (CTAFFNet) for object detection in complex traffic scenarios.

### 2.3. Perception Based on CNN and Transformer

To fully leverage the advantages of both CNN and Transformer, researchers have proposed various methods to combine them [32,33]. A common strategy is to use a CNN to extract local features of images and then feed these features into a Transformer for global information modeling. Another strategy is to stack CNN and Transformer in parallel and integrate their features through a fusion mechanism [25]. Such hybrid architectures have achieved good performance in various image perception tasks. FATCNet [30] proposed a Feature Adaptive Transformer and CNN for infrared small target detection. MSFCTNet [20] proposed a Multi-Scale Fusion CNN–Transformer Network for high-resolution remote sensing image change detection, aiming to improve change detection capability. Bridging CNN and Transformer [25] proposed a cross-attention fusion network for hyperspectral image classification. Improving RGB–infrared object detection [29] proposed a Cascaded Alignment-Guided Transformer. A Transformer-encoder-based multi-modal multi-attention fusion network [34] proposed a network for sentiment analysis. Transformer–CNN [35] proposed a network for small image object detection. Ref. [36] proposed a variant vision transformer for automatic localization of polyps in gastroscopic images.

## 3. Methods

### 3.1. Overall Framework

The proposed framework is designed to address the dual objectives of high-fidelity image fusion and robust object detection through a unified trainable architecture. Let $I_{ir}\in R^{H\times W\times C_{in}}$ and $I_{vi}\in R^{H\times W\times C_{in}}$ denote the aligned infrared and visible source images, respectively, and let $I_{f}\in R^{H\times W\times C_{out}}$ denote the fused image. Here, *H*, *W*, $C_{in}$, and $C_{out}$ represent the image height, width, input-channel number, and output-channel number, respectively. The objective is to aggregate complementary local and global information from the two modalities and improve scene perception for detection.

**Figure 2:**
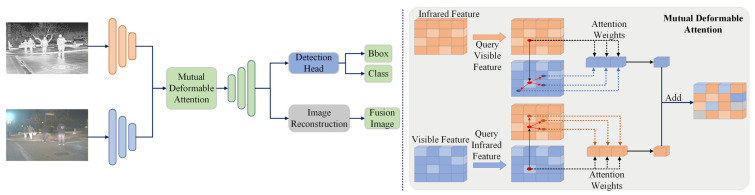
An overview of the proposed framework.

Based on the parallel principle, our proposed framework is shown in Figure 2. It is divided into four parts: Feature Extraction, Mutual Deformable Attention, Object Detection, and Image Reconstruction modules. This fully enables the perception of fine-grained detail information and the effective extraction of semantic information. The image fusion network generates the fused image, while the object detection network provides semantic features. Both tasks jointly constrain the network, enhancing the model’s robustness and interpretability.

### 3.2. Multi-Scale Feature Extraction

Unlike standard single-scale extraction, we employ a hierarchical encoder to capture semantic information at varying resolutions. The infrared image $I_{ir}$ and visible image $I_{vi}$ are fed into parallel backbone networks (e.g., ResNet or Swin Transformer variants) to extract multi-scale features. The feature extraction process can be formulated as

(1) $$\left\{F_{ir}^{l},F_{vi}^{l}\}=\{H_{enc}^{l}(I_{ir}),H_{enc}^{l}(I_{vi})\},l\in \{1,2,3\right\}$$

This provides a simple and effective way to extract local semantic information and map local information to a high-dimensional feature space. This process generates feature maps at resolutions of $\frac{1}{2},\frac{1}{4}$ and $\frac{1}{8}$ of the input size. These multi-scale features provide rich local semantic context and are subsequently fed into the Mutual Deformable Attention module for cross-modal interaction.

### 3.3. Mutual Deformable Attention

**Figure 3:**
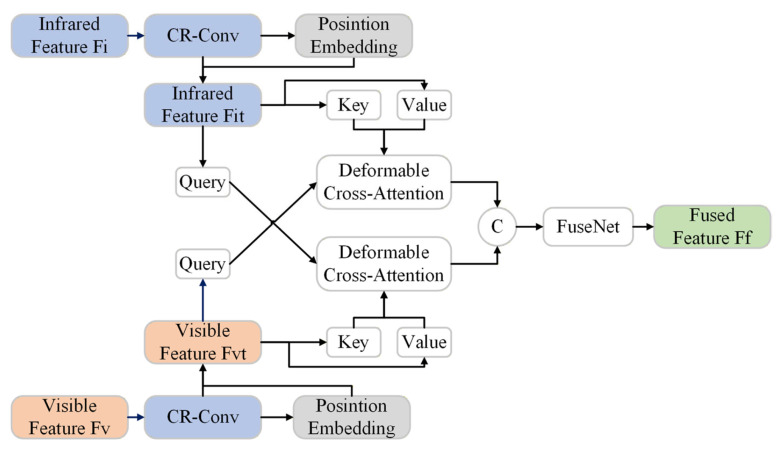
Structure of the Mutual Deformable Attention module.

To mitigate spatial interference caused by noise and data-corrupted regions, this paper proposes a Mutual Deformable Attention (MDA) module (illustrated in Figure 3) for effectively integrating discriminative features from infrared and visible modalities. Unlike conventional attention mechanisms that traverse all spatial positions, MDA aggregates features from only a small set of key sampling points near the reference query, with attention weights adaptively assigned to each position.

Specifically, let $F_{i}$ and $F_{v}$ denote the infrared and visible feature tensors at the l-th scale, respectively, where $F_{i},F_{v}\in R^{C_{d}\times \frac{H}{S}\times \frac{W}{S}}$. The feature enhancement is achieved through a bidirectional interaction mechanism, i.e., Infrared-to-Visible and Visible-to-Infrared interactions:

(2) $$\begin{array}{l} Infrared2Visible:F_{i}^{’}\leftarrow MulDeAttn(q,F_{i})\\ Visible2Infrared:F_{v}^{’}\leftarrow MulDeAttn(q,F_{v}) \end{array}$$

For fine-grained fusion of multi-modal features, $F_{Q}$ denotes the query feature at the reference spatial position $p_{q}$. The deformable attention applied to the infrared feature map is calculated as follows:

(3) $$DeformAttn(q,F_{i})=\sum_{k=1}^{K}A_{k}\times W^{V}F_{i}(p_{q}+\Delta p_{k})$$

Here, *K* is the number of sampled positions. $\Delta p_{k}$ and $A_{k}$ denote the learned offset and attention weight for the *k*-th sampling point, respectively, and the weights are normalized as follows:

(4) $$\sum_{k=1}^{K}A_{k}=1$$

The sampling offset $\Delta p_{k}$ and attention weight $A_{k}$ are predicted from the query *q* by two independent linear layers. The operator $F_{i}(p_{q}+\Delta p_{k})$ denotes feature sampling at the shifted position, implemented with bilinear interpolation when the coordinates are fractional. We employ a multi-head attention mechanism:

(5) $$MulDeAttn(q,F_{i})=\sum_{m=1}^{M}W_{m}DeformAttn(q,F_{i})$$

where *M* denotes the number of attention heads and $W_{m}$ is the learnable projection matrix of the *m*-th head. Similarly, the deformable attention for the visible feature $F_{v}$ is formulated as

(6) $$DeformAttn(q,F_{v})=\sum_{k=1}^{K}A_{k}\times W^{V}F_{v}(p_{q}+\Delta p_{k})$$

(7) $$MulDeAttn(q,F_{v})=\sum_{m=1}^{M}W_{m}DeformAttn(q,F_{v})$$

Finally, the fused feature $F_{f}$ is aggregated at spatial positions as

(8) $$F_{f}(p_{q})=q+MulDeAttn(q,F_{i})+MulDeAttn(q,F_{v})$$

Thus, the model adaptively aggregates features at informative positions for each modality, which can reduce the influence of corrupted or low-quality regions rather than completely eliminate it.

### 3.4. Image Reconstruction

Upon aggregating complementary information from multi-modal sources, the fused deep features require a sophisticated decoding strategy to restore spatial fidelity. To achieve this, we devise a hierarchical reconstruction framework comprising a Transformer-based Deep Feature Reconstruction unit $H_{DR}$ and a CNN-based Image Reconstruction unit $H_{IR}$. This design synergizes the global modeling capability of Transformers with the local texture synthesis strength of CNNs: $H_{DR}$ refines the fused features from a global perspective to ensure semantic consistency, while $H_{IR}$ recovers high-frequency shallow details to generate the final image.

Specifically, the $H_{DR}$ module incorporates three Swin Transformer layers to capture long-range dependencies, followed by the $H_{IR}$ module, which employs three convolutional layers to progressively reduce feature channels and synthesize the fused image $I_{f}$. This cascaded reconstruction process is formulated as

(9) $$I_{f}=H_{IR}(H_{DR}(F_{f}))$$

where the kernel size of the convolutional layers is 3 × 3 with a stride of 1.

### 3.5. Loss Function

The overall training objective of our proposed framework is driven by a joint loss function that balances the quality of the fused image and the accuracy of the object detection. As illustrated in Figure 1, our network consists of a multi-modal fusion module followed by two parallel branches: an Image Reconstruction branch for generating the fused image, and a Detection Head for predicting object categories and locations. Accordingly, the total loss function total $L_{total}$ is formulated as a weighted sum of the image fusion loss $L_{f}$ and the detection-driven loss $L_{d}$:

(10) $$L_{total}=\lambda_{fus}\times L_{f}+\lambda_{det}\times L_{d}$$

where $\lambda_{fus}$ and $\lambda_{det}$ are hyperparameters used to control the trade-off between the visual quality of the fusion and the detection performance.

#### 3.5.1. Image Fusion Loss

To ensure the fused image retains rich structural, textural, and intensity information from the source images (infrared and visible), the fusion loss $L_{f}$ is composed of three specific sub-loss terms:

(11) $$L_{f}=\alpha \times L_{ssim}+\beta \times L_{text}+\gamma \times L_{int}$$

SSIM Loss ($L_{ssim}$): To preserve the structural similarity between the fused image $I_{f}$ and the source images $I_{vis}$ and $I_{ir}$, we employ the SSIM loss. It constrains the network to maintain luminance, contrast, and structure consistency:

(12) $$L_{ssim}=w_{1}\times (1-ssim(I_{f},I_{1}))+w_{2}\times (1-ssim(I_{f},I_{2}))$$

where ssim(⋅) denotes the structural similarity operation. In practice, we set $w_{1}=w_{2}=0.5$.

Texture Loss ($L_{text}$): To effectively aggregate texture details from the source images into a single fused image, the texture loss $L_{text}$ is defined as

(13) $$L_{text}=\frac{1}{HW}{\left\|\left|\nabla I_{f}\right|-\max(\left|\nabla I_{1}\right|,\left|\nabla I_{2}\right|)\right\|}_{1}$$

where ∇ denotes the Sobel gradient operator, |⋅| denotes the absolute operation, ${\left\|\cdot \right\|}_{1}$ denotes the $L_{1}$ norm, and max(⋅) denotes the element-wise maximum selection.

Intensity Loss ($L_{int}$): To appropriately fuse the global intensity information of the source images, an intensity loss function is used to constrain the network model. The intensity loss is defined as

(14) $$L_{\mathrm{int}}=\frac{1}{HW}{\left\|I_{f}-M(I_{1},I_{2})\right\|}_{1}$$

where M(·) is the element-wise aggregation operation.

#### 3.5.2. Detection-Driven Loss

Different from traditional fusion methods that focus solely on image quality metrics, our framework introduces a Detection Head (as shown in Figure 1) to provide semantic feedback. The detection-driven loss $L_{d}$ is calculated based on the output of this head. Following the standard object detection protocols (e.g., YOLO series), $L_{d}$ consists of classification loss and regression loss:

(15) $$L_{d}=L_{cls}+\lambda_{reg}\times L_{reg}$$

Classification Loss ($L_{cls}$): We utilize the Cross-Entropy loss to minimize the discrepancy between the predicted probability distribution and the ground-truth labels. This forces the fusion network to enhance features relevant to object categories.

Regression Loss ($L_{reg}$): We adopt the Smooth L1 loss to optimize the bounding box coordinates, ensuring precise localization of objects within the fused scene.

By minimizing $L_{d}$, the gradients are back-propagated to the multi-modal fusion module, effectively guiding it to generate a fused representation that maximizes the separability of object features, thus achieving a detection-driven fusion strategy.

## 4. Experiments

### 4.1. Experimental Settings

#### 4.1.1. Datasets

To comprehensively evaluate the performance and generalization capability of the proposed method across diverse scenarios, we conduct experiments on five widely used public infrared–visible image datasets: M3FD [5], LLVIP [9], TNO [37], RoadScene [38], and MSRS [39]. These datasets cover a wide range of scenes from daily driving to extreme environments, providing a solid foundation for multi-modal fusion and detection research.

Analysis of Dataset Characteristics and Selection Rationale: M^3^FD [5] serves as the primary evaluation benchmark in this study. It is not only one of the larger, well-annotated infrared–visible detection datasets available but, more importantly, it encompasses eight different scene types (e.g., road, campus, smoke, forest) and various extreme imaging conditions (e.g., glare, rain, fog). This diversity allows us to systematically assess the model’s robustness and generalization against scene distribution shifts and complex environmental interference, moving beyond overfitting to a single ideal scenario. LLVIP [9] is specifically constructed for low-light vision tasks. In this dataset, visible images lose substantial texture and color information due to insufficient illumination, which acutely amplifies the complementary value of the infrared modality. Evaluating on LLVIP directly tests whether our fusion framework can effectively extract and integrate crucial thermal radiation information from infrared images to maintain detection performance when the visible modality is nearly ineffective. TNO [37] and RoadScene [38] are classic benchmarks in the infrared–visible image fusion field. Although they lack object detection annotations, they provide a large number of high-quality, registered image pairs. Conducting qualitative visual comparisons and quantitative fusion metric evaluations (e.g., EN, MI, VIF) on these datasets helps verify that our proposed method surpasses existing state-of-the-art methods in terms of pixel-level information fusion quality, ensuring the visual credibility of the fusion results. MSRS [39] provides not only image pairs but also semantic segmentation labels. This allows for additional task-driven analysis, such as investigating whether the fused images can improve semantic segmentation accuracy. This helps argue that the features generated by our method possess good transferability to multiple high-level vision tasks.

Discussion of Limitations: While the aforementioned datasets constitute a comprehensive test suite, they share certain common limitations that may affect the assessment of a model’s true generalization capability: (a) Scene Bias: The data is predominantly focused on road surveillance and driving perspectives, leaving generalization to other critical application domains (e.g., medical imaging, remote sensing) unverified; (b) Limited Object Categories: Annotations concentrate on a few common categories (e.g., person, car), providing insufficient examination of detection capability for rare or fine-grained objects; (c) Simulation-to-Reality Gap: Some adverse weather scenes (e.g., rain and fog in M^3^FD) may be simulated or limited, differing from the continuous, complex physical degradation processes in the real world. Future benchmark development should aim for broader application domains and more realistic physical degradation modeling.

#### 4.1.2. Evaluation Metrics

To comprehensively evaluate the object detection performance and practical applicability of the proposed method, we adopt a multi-dimensional evaluation protocol encompassing accuracy, efficiency, and model complexity.

1. Detection Accuracy Metrics

We employ the widely recognized mean Average Precision (mAP) as the primary accuracy metric. The Average Precision (AP) for a single category is defined as the area under its precision–recall (P-R) curve:

(16) $$AP=\int_{0}^{1}P(r)dr$$

where P(r) denotes precision at recall level r. To assess performance under different localization strictness, we report: (1) mAP@50: The mean AP across all categories at an Intersection over Union (IoU) threshold of 0.50; (2) mAP@75: The mean AP across all categories at a stricter IoU threshold of 0.75; (3) mAP: The primary metric, calculated as the average AP for IoU thresholds ranging from 0.50 to 0.95 in steps of 0.05. This provides a holistic view of the detector’s performance across varying overlap criteria [40].

2. Model Efficiency and Complexity Metrics

For real-world deployment, especially in latency-sensitive applications like autonomous driving, computational efficiency is crucial. Therefore, we supplement accuracy evaluation with the following metrics: (1) Processing Speed (FPS): Measured in frames per second on a standard GPU (NVIDIA RTX 3090, Santa Clara, CA, USA), representing the average throughput for processing a pair of visible and infrared images. Higher FPS indicates better suitability for real-time systems. (2) Model Parameters (Params): The total number of trainable parameters, reported in millions (M). This reflects the model’s memory footprint and storage requirement. (3) Computational Cost (FLOPs): The number of floating-point operations required for a single forward pass, reported in gigaFLOPs (G). This quantifies the theoretical computational burden, closely related to energy consumption.

These efficiency metrics allow for a direct comparison with state-of-the-art methods regarding the trade-off between accuracy and resource consumption, which is essential for assessing practical utility [41].

#### 4.1.3. Implementation Details

All experiments are conducted using the PyTorch 3.8 framework. The model is optimized with Stochastic Gradient Descent (SGD) over 100 epochs with a batch size of 6. The hyperparameters that control the trade-off of each sub-loss term are empirically set as α = 10, β = 20, and γ = 20. Considering the computational cost of repeated training on multiple datasets, the current revision reports single-run results under fixed settings; therefore, the corresponding claims are limited to empirical benchmark comparisons rather than formal statistical significance.

### 4.2. Comparison with State-of-the-Art Models

#### 4.2.1. Qualitative Comparison

To ensure comprehensiveness and fairness in the evaluation, this paper systematically evaluated different image fusion methods based on extensive qualitative visualization experiments across multiple public datasets. We selected current representative image fusion methods—IFCNN [3], SwinFusion [42] and PIAFusion [43]—for comparative experiments. Their fused visible–infrared image pairs were generated and further evaluated for performance in downstream object detection tasks. To ensure fairness and consistency, all fused images were processed using the YOLOv8 [44] object detection model, which also serves as the backbone of our proposed method. It should be noted that for some single-stage detection methods, object detection can be performed directly on the original modal images without prior image fusion. Therefore, in relevant experiments, we concatenated the original visible and infrared images and overlaid the detection results on the combined image for more intuitive result analysis and comparison.

**Figure 4:**
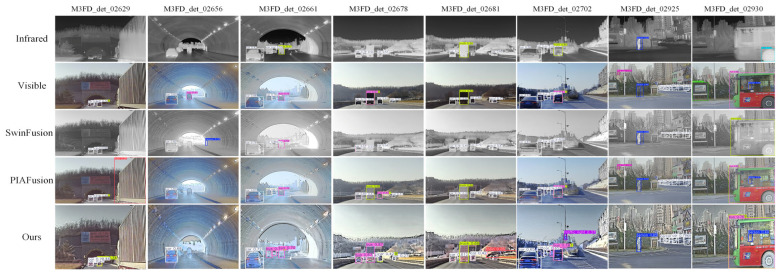
Qualitative comparison of different methods in the M3FD_Detection dataset.

**Figure 5:**
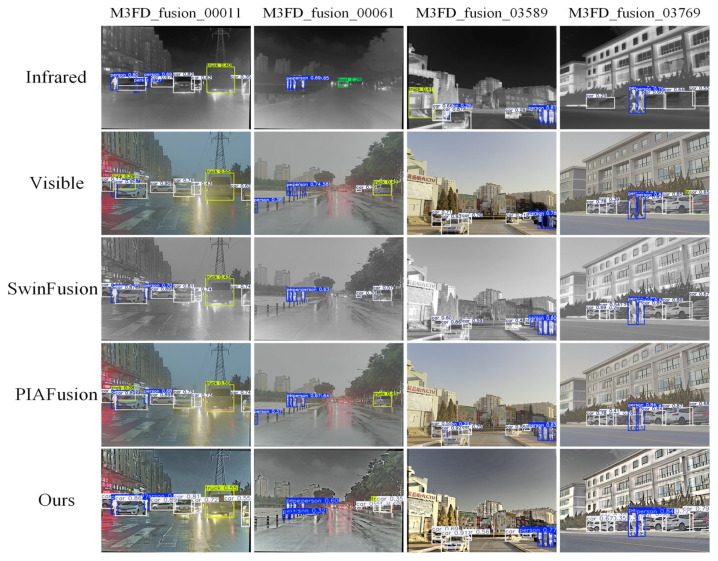
Qualitative comparison of different methods in the M3FD_Fusion dataset.

**Figure 6:**
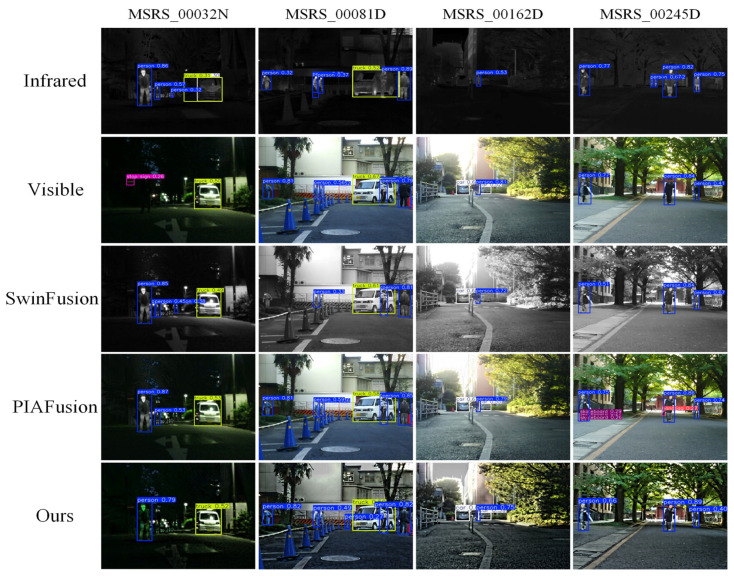
Qualitative comparison of different methods in the MSRS dataset.

**Figure 7:**
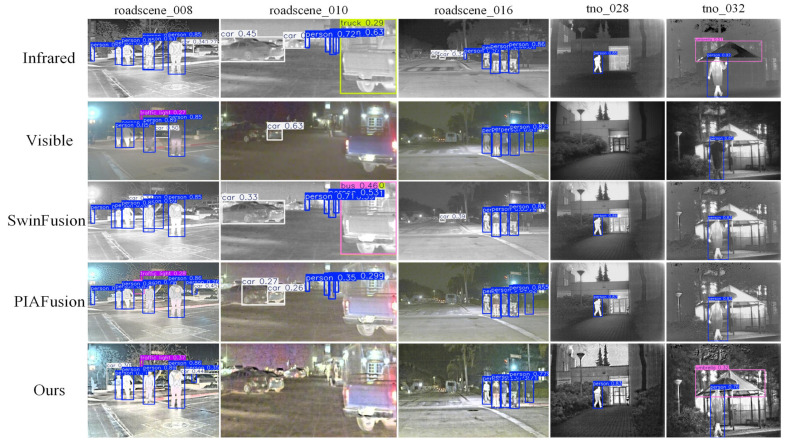
Qualitative comparison of different methods in the RoadScene and TNO dataset.

To further verify the adaptability of the proposed method under different weather conditions, we conducted extended experiments on the M3FD [5] dataset, with relevant results shown in Figure 4 and Figure 5. This dataset includes several practically relevant complex-weather scenes. For example, under haze and smoke conditions, the visible modality is usually severely disturbed, making it difficult to provide clear target information, while the infrared modality has stronger penetration and target visibility. Therefore, fully utilizing the complementarity between the two modalities is particularly crucial for improving human body detection performance in smoke environments. However, under extreme conditions such as uneven smoke distribution and complex lighting, some fusion methods show significant deficiencies in target edge and detail extraction. In contrast, the proposed method uses an interactive Deformable Transformer module to capture target detail features, edge information, and contour structures, showing advantages in low-light, foggy, and other complex weather scenes. By enhancing the modeling ability of target details, it improves the perception and localization accuracy of objects in complex scenes, thereby achieving a deeper understanding of the differences and correlations between visible and infrared modalities, thereby improving detection accuracy in the evaluated scenes. To comprehensively evaluate the adaptability and generalization capability of the proposed method, we also conducted empirical tests on multiple multi-spectral datasets including MSRS, TNO, and RoadScene (results shown in Figure 6 and Figure 7). The visual comparisons suggest that the proposed method, by introducing the Mutual Deformable Transformer module and multi-task training constraints, can enhance the perception of target information at different spatial positions in visible and infrared modalities, further strengthening its edge and structural feature expression capabilities, indicating favorable robustness and detection accuracy in multi-modal fusion object detection tasks.

The proposed framework may still struggle when both modalities are severely degraded, e.g., dense smoke combined with low thermal contrast, strong thermal clutter, severe target occlusion, or imperfect VIS–IR registration. In such cases, the attention module may sample from ambiguous background regions, leading to missed detections of small pedestrians or inaccurate bounding boxes around partially visible vehicles. These cases indicate that the current method should be regarded as a robust architectural integration for common low-light and complex-weather scenes, rather than a complete solution for all adverse environments.

#### 4.2.2. Quantitative Comparison

To ensure comprehensiveness and fairness in the evaluation, this paper utilized various image fusion methods, including PIAFusion [43] and SwinFusion [42], and selected current mainstream single-modal object detectors YOLOv5 and YOLOv7 [40] for performance comparison.

Table 1 shows the systematic quantitative comparison of various models on the FLIR dataset, using key performance metrics such as mAP@50 commonly used in object detection tasks. The table is divided into two parts, “single-modal models” and “multi-modal models”, to clearly present the detection capabilities of different methods under single-modal and multi-modal conditions. In the single-modal part, we evaluated the detection performance of YOLOv5 and YOLOv7 on visible (VIS) and infrared (IR) modalities separately. Taking YOLOv5 as an example, the model equipped with the CSPDarknet backbone achieved 67.8% mAP@50 under VIS modality and increased to 73.9% under IR modality, indicating significant differences in the model’s perception ability of targets under different modalities. In the multi-modal part, we focused on analyzing image fusion-based detection methods like PIAFusion and SwinFusion. Experimental results show that these methods generally outperform single-modal methods after fusing VIS and IR information, demonstrating stronger perception and generalization capabilities. For instance, the fusion model achieved 77.8% mAP@50 on the FLIR dataset, higher than the single-modal detection results, supporting the effectiveness of multi-modal fusion in improving object detection performance. Overall, multi-modal methods achieved better performance than single-modal methods on the FLIR dataset, especially obtaining higher mAP@50 in detection accuracy. It is worth noting that SwinFusion and PIAFusion have slight advantages in mAP@75 on FLIR, which may stem from aggressive fusion strategies for texture details under low-resolution inputs, while our method achieves the best performance in mAP@50, reflecting more robust general object localization capability while preserving key thermal features. This result further supports the effectiveness and practical value of our proposed multi-modal fusion detection framework in visible–infrared (VIS-IR) object detection tasks.

Table 2 shows the performance comparison results of various models on the M3FD [5] dataset. First, in the single-modal part, we evaluated two mainstream object detection methods: YOLOv5 and YOLOv7. Under visible (VIS) or infrared (IR) modalities, the mAP@50 scores of these two methods range between 66.9% and 70.8%, indicating good detection performance under a single modality. Furthermore, in the multi-modal detection part, we compared several single-stage fusion methods, including the detection effects of YOLOv5 and YOLOv7 after fusing VIS and IR images. The results show that multi-modal input significantly improves detection performance. Particularly noteworthy, the method proposed in this paper achieves the best results on both mAP@50 and mAP@75 metrics, reaching 74.2% and 42.1% respectively. This not only indicates that this method has strong detection capability in the challenging multi-modal detection scenario of the M3FD [5] dataset but also suggests its good computational efficiency and model complexity control while ensuring high detection accuracy.

Table 3 systematically compares the performance of various fusion models on the LLVIP dataset, covering single-stage and two-stage detection methods that fuse VIS and IR images. The experiment employed multiple evaluation metrics, including mAP@50 and mAP@75, to comprehensively evaluate the detection effectiveness of each method in low-light environments. Among all multi-modal methods, the method proposed in this paper performs particularly well, achieving mAP@50 and mAP@75 of 98.6% and 73.4% respectively, outperforming the other comparison models under the reported setting. These results support the advantage of our method in detecting targets like pedestrians under low-light conditions, showcasing its efficient utilization of complementary information between visible and infrared images. In summary, the proposed method demonstrates excellent performance on three representative datasets—FLIR, M3FD, and LLVIP—supporting its effectiveness and robustness in multi-modal object detection tasks.

#### 4.2.3. Quantitative Analysis of Model Efficiency and Complexity

To comprehensively assess the practical potential of the proposed method, this section provides a quantitative analysis from the perspectives of model efficiency and computational complexity. As shown in Table 4, we compare the proposed method with two advanced baseline methods on three key metrics: Number of Parameters, Computational Cost (FLOPs), and Processing Speed (FPS).

Proposed Method vs. Baselines: (1) Parameters (M): Ours (3.25) > SwinFusion (3.09) > PIAFusion (1.18). Our higher count stems from the added deformable attention and detection head modules, trading parameter efficiency for enhanced feature interaction and task awareness. (2) FLOPs (G): PIAFusion (77.01) > SwinFusion (40.57) > Ours (18.50). Despite more parameters, our method achieves the lowest computational cost, benefiting from the efficient sparse computation of deformable attention and an optimized architecture. (3) Speed (FPS): PIAFusion (117.89) > Ours (4.50) > SwinFusion (1.90). Our inference speed is over twice that of SwinFusion, balancing performance with practical latency tolerance for applications like surveillance, though slower than the highly optimized PIAFusion.

Summary: Our method achieves the lowest FLOPs among the compared methods and faster inference than the Transformer-based SwinFusion baseline. It suggests a favorable balance between model capacity, computational burden, and runtime, supporting its core detection-aware fusion design.

### 4.3. Ablation Study

An ablation study was conducted on the M3FD [5] dataset, with the results presented in Table 5. Starting from a baseline detection model, we sequentially introduced the Multi-scale Feature Extraction (MFE) module, the Mutual Deformable Attention (MDA) module, and two sub-terms of the multi-task loss function—the feature-fusion loss term $L_{f}$ and the object-detection loss term $L_{d}$—to progressively evaluate the contribution of each component to the overall performance.

The following observations can be drawn from the table:

(1) Baseline (Row 1): Without any enhancement modules, the model achieved 60.3% mAP@50 and 33.9% mAP@75, establishing a performance baseline for subsequent comparisons.
(2) Adding the MFE module (Row 2): mAP@50 increased to 64.1% and mAP@75 to 34.3%, indicating that Multi-scale Feature Extraction improves the model’s ability to capture target contours and edges across different scales, especially in complex backgrounds.
(3) Further introducing the MDA module (Row 3): mAP@50 rose substantially to 68.9% and mAP@75 to 40.1%, demonstrating that MDA effectively guides the model to learn complementary features between visible and infrared modalities, thereby enhancing the quality of multi-modal information fusion.
(4) Introducing the feature-fusion loss $L_{f}$ (Row 4): mAP@50 further improved to 71.5% and mAP@75 to 41.4%, confirming that this loss term plays a significant role in optimizing cross-modal feature alignment and reducing information redundancy caused by inter-modal discrepancies.
(5) Introducing the object-detection loss $L_{d}$ (Row 5): The final configuration reached 74.2% mAP@50 and 42.1% mAP@75, yielding the best performance among all settings. This indicates that the discriminative enhancement constraint further strengthens the model’s ability to distinguish targets from the background, leading to higher detection accuracy, particularly in complex scenes.

The ablation study indicates that each major component contributes incremental performance gains under the tested setting, supporting the usefulness of the Multi-scale Feature Extraction module, the Mutual Deformable Attention module, and the two loss terms. Nevertheless, the current ablation focuses on major modules and the principal loss components. A more exhaustive analysis of hyperparameter sensitivity and fine-grained module interactions is left for future work.

In particular, the transition from Row 2 to Row 3 reflects the interaction between multi-scale features and deformable attention: the MFE module provides multi-resolution local cues, while the MDA module selectively samples cross-modal complementary information. Rows 4 and 5 further indicate that the image-fusion loss mainly improves cross-modal feature alignment, whereas the detection-driven loss encourages features that are more discriminative for object localization and classification. These observations are explanatory rather than a substitute for a full sensitivity analysis.

To further verify robustness under low-light conditions, we conduct comparative experiments on two representative low-light multi-modal datasets: M3FD [5] and LLVIP [9]. In such scenes, infrared images significantly accentuate the thermal-radiation characteristics of human targets, serving as a critical supplement when visible-image quality severely deteriorates. Unlike existing methods that often suffer from high false-alarm rates and severe missed detections, our proposed Mutual Deformable Transformer architecture and multi-task learning mechanism fully exploit complementary information between modalities, more effectively aligning their spatial and semantic features to improve detection accuracy.

**Figure 8:**
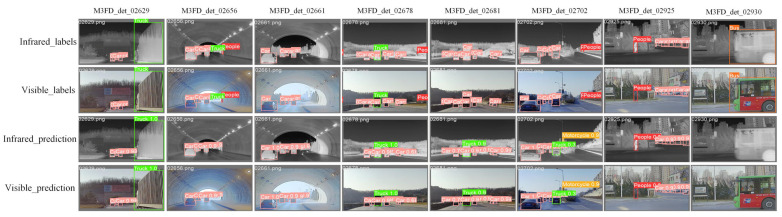
Detection results of the proposed method in the M3FD_Detection dataset.

**Figure 9:**
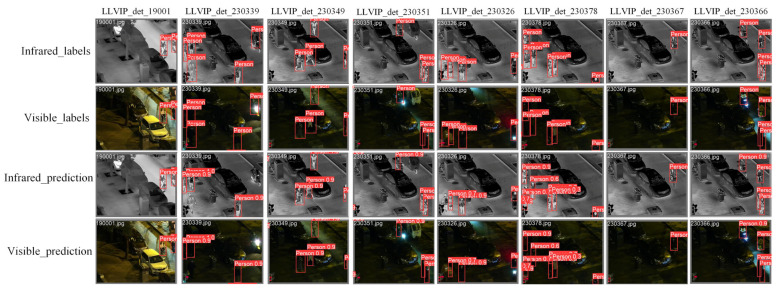
Detection results of the proposed method in the LLVIP dataset.

Specifically, the Mutual Deformable Attention module enhances perception of target contours and edges, enabling stable identification even in low-light or cluttered backgrounds. The multi-task learning strategy improves feature discriminability and detection robustness through auxiliary constraints. Figure 8 illustrates accurate detection results on M3FD under complex weather and low-light conditions, while Figure 9 showcases strong performance on LLVIP in nighttime and extremely dark environments, verifying the method’s superior adaptability across a range of low-illumination scenes.

In conclusion, the experiments indicate that the proposed method exhibits favorable robustness under the evaluated low-light conditions, enhancing the practicality and reliability of multi-modal detection systems with broad application prospects.

## 5. Conclusions

This paper addresses several key challenges of infrared–visible image fusion in object detection tasks and proposes a multi-modal fusion detection framework based on the combination of Convolutional Neural Network (CNN) and Deformable Transformer. This framework comprehensively utilizes CNN’s ability for precise modeling of local details and Transformer’s advantages in capturing global dependency information, showing performance improvements in image feature extraction, modal complementary information fusion, and downstream detection tasks.

Specifically, we designed a detection-aware fusion mechanism, realizing joint optimization of the fusion process and detection objectives, which helps alleviate the information bottleneck problem inherent in traditional “fuse-then-detect” methods. Simultaneously, by introducing a dynamic modal weighting module, the model can adaptively adjust the importance weights of infrared and visible features based on scene semantics, achieving more precise feature fusion. Furthermore, addressing the scarcity of training samples for infrared images, this paper proposed a multi-spectral pre-training strategy, which aims to improve the model’s robustness and generalization capability in real complex environments.

Extensive experiments on multiple challenging multi-modal datasets, including M3FD and LLVIP, supported the effectiveness of the proposed method. The results show that the proposed model achieves competitive performance compared to existing mainstream methods in both object detection accuracy and robustness, with advantages under adverse conditions such as low-light and complex backgrounds.

Our method has limitations that suggest promising research avenues: (1) Small and Occluded Targets: Accuracy for small or heavily occluded objects needs improvement. Future work may involve multi-scale feature pyramids or occlusion-aware mechanisms. (2) Model Efficiency: The CNN–Transformer design increases complexity. Exploring lightweight Transformers, model compression, or efficient attention can aid edge deployment. (3) Broader Generalization: Validation is currently limited to road/surveillance scenes. Extending to medical image fusion, multi-focus fusion, and other downstream tasks (e.g., semantic segmentation) is warranted. (4) Hyperparameter Adaptation: The joint loss is sensitive to weight balancing. Automatically determining hyperparameter proportions would enhance robustness and reduce tuning effort. (5) Limited Statistical Validation and Ablation Depth: The current experiments report single-run benchmark results without confidence intervals or significance tests, and the ablation focuses on major components and principal loss terms. Future work should include repeated runs, confidence intervals, and more comprehensive analyses of hyperparameter sensitivity, loss contributions, and module interactions. (6) Failure Cases: Performance may degrade under extreme illumination, heavy noise, severe occlusion, thermal clutter, or inaccurate cross-modal registration. A systematic failure-case benchmark remains an important direction for future work.

In conclusion, this work presents a practical framework for infrared–visible image fusion that is cognizant of downstream detection needs. It offers an empirical reference for enhancing multi-modal perception systems in applications like autonomous driving and intelligent surveillance. Future efforts will focus on overcoming the aforementioned limitations to achieve more robust, efficient, and versatile multi-modal visual understanding.

## Figures and Tables

**Table 1:** Quantitative comparison of different methods on the FLIR dataset.

	Modality	mAP50 (%)	mAP75 (%)
YOLOv5	VIS	67.8	25.9
YOLOv5	IR	73.9	35.7
YOLOv7	VIS	70.2	32.7
YOLOv7	IR	75.6	32.2
IFCNN	VIS + IR	73.8	34.9
SwinFusion	VIS + IR	74.2	35.8
PIAFusin	VIS + IR	75.3	35.7
Ours	VIS + IR	77.8	34.9

**Table 2:** Quantitative comparison of different methods on the M3FD dataset.

	Modality	mAP50 (%)	mAP75 (%)
YOLOv5	VIS	66.9	37.8
YOLOv5	IR	68.7	39.2
YOLOv7	VIS	69.3	38.1
YOLOv7	IR	70.8	40.7
IFCNN	VIS + IR	67.3	38.7
SwinFusion	VIS + IR	68.9	40.5
PIAFusin	VIS + IR	69.9	41.6
Ours	VIS + IR	74.2	42.1

**Table 3:** Quantitative comparison of different methods on the LLVIP dataset.

	Modality	mAP50 (%)	mAP75 (%)
YOLOv5	VIS	90.8	51.9
YOLOv5	IR	94.6	72.2
YOLOv7	VIS	91.9	52.9
YOLOv7	IR	96.0	72.9
IFCNN	VIS + IR	94.8	71.4
SwinFusion	VIS + IR	95.2	72.3
PIAFusin	VIS + IR	96.1	72.6
Ours	VIS + IR	98.6	73.4

**Table 4:** Quantitative comparison of efficiency and complexity for different methods.

	Parameters (M)	FLOPs (G)	Speed (FPS)
SwinFusion	3.09	40.57	1.90
PIAFusion	1.18	77.01	117.89
Ours	3.25	18.50	4.50

Note: All FPS measurements are conducted on an NVIDIA RTX 3090 GPU with an input image resolution of 256×256 and a batch size of 1.

**Table 5:** Ablation study on the M3FD dataset.

Index	Baseline	MFE	MDA	$L_{f}$	$L_{d}$	mAP50 (%)	mAP75 (%)
1	√					60.3	33.9
2	√	√				64.1	34.3
3	√	√	√			68.9	40.1
4	√	√	√	√		71.5	41.4
5	√	√	√	√	√	74.2	42.1

Note: The columns $L_{f}$ and $L_{d}$ denote the image fusion loss and the detection-driven loss defined in Equation (10), respectively. √ denotes that this entry introduces the corresponding module.

## Data Availability

The original contributions presented in this study are included in the article. Further inquiries can be directed to the corresponding author.
